# Regional Microstructural Integrity in Relation to Gait Speed: The Atherosclerosis Risk in Communities Study

**DOI:** 10.1093/geroni/igab046.275

**Published:** 2021-12-17

**Authors:** Kevin Sullivan, Chad Blackshear, Timothy Hughes, Rebecca Gottesman, Prashanthi Vemuri, Thomas Mosley, Michael Griswold, B Gwen Windham

**Affiliations:** 1 The University of Mississippi Medical Center, Jackson, Mississippi, United States; 2 University of Mississippi Medical Center, Jackson, Mississippi, United States; 3 Wake Forest School of Medicine, Winston-Salem, North Carolina, United States; 4 Johns Hopkins University, Baltimore, Maryland, United States; 5 The Mayo Clinic, Mayo Clinic, Minnesota, United States

## Abstract

Brain imaging-based biomarkers of neuropathology are associated with mobility in older adults, but the relation of regional microstructural integrity to gait speed in the context of a broader neuropathological profile is less understood. We examined cross-sectional associations of microstructural integrity with 4-meter usual-pace gait speed (cm/s) in a subsample of ARIC study participants who completed 3T MRI brain scans with diffusion tensor imaging(2011-13; n=1785; mean age=76.2±5.3, 60% Female, 28% Black). We considered total brain and six regional averages of fractional anisotropy (FA; lower=worse microstructural integrity) and mean diffusivity (MD; higher=worse microstructural integrity): frontal, temporal, parietal, occipital, anterior and posterior corpus callosum. Associations were tested in multivariable linear regression models adjusted for demographics, cardiovascular risk factors, and with and without additional neuropathological indices: total brain volume, white matter hyperintensities, infarcts, and microhemorrhages. When modeled separately, all neuropathology indices were associated with slower gait speed. Every standard deviation(SD) higher total brain FA was associated with +2.56 cm/s gait speed (95%CI: 1.64,3.48) and every SD higher MD was associated with -4.27 cm/s gait speed (-5.34,-3.20). All regional estimates were comparable. When adjusted for all other neuropathology indices, only posterior corpus callosum FA (β=1.72; 0.67,2.77), total MD (β=-1.63; -3.02,-0.25), frontal lobe MD (β=-1.76; -3.03,-0.48), and temporal lobe MD (β=-1.40; -2.78,-0.02) remained significantly associated with gait speed. Microstructural integrity is an informative measure of brain pathology in relation to mobility, with regional measures tied to executive, memory, and somatosensory function being more informative when a broader neuropathological profile is considered.

